# Dietary Supplement Use among U.S. Children by Family Income, Food Security Level, and Nutrition Assistance Program Participation Status in 2011–2014

**DOI:** 10.3390/nu10091212

**Published:** 2018-09-01

**Authors:** Shinyoung Jun, Alexandra E. Cowan, Janet A. Tooze, Jaime J. Gahche, Johanna T. Dwyer, Heather A. Eicher-Miller, Anindya Bhadra, Patricia M. Guenther, Nancy Potischman, Kevin W. Dodd, Regan L. Bailey

**Affiliations:** 1 Department of Nutrition Science, Purdue University, West Lafayette, IN 47907, USA; jun24@purdue.edu (S.J.); cowan9@purdue.edu (A.E.C.); heicherm@purdue.edu (H.A.E.-M.); 2 Department of Biostatistical Sciences, School of Medicine, Wake Forest University, Winston-Salem, NC 27157, USA; jtooze@wakehealth.edu; 3 Office of Dietary Supplements, National Institutes of Health, Bethesda, MD 20892-7517, USA; jaime.gahche@nih.gov (J.J.G.); dwyerj1@od.nih.gov (J.T.D.); potischn@mail.nih.gov (N.P.); 4 Department of Statistics, Purdue University, West Lafayette, IN 47907, USA; bhadra@purdue.edu; 5 Department of Nutrition and Integrative Physiology, University of Utah, Salt Lake City, UT 84112, USA; patricia.guenther@utah.edu; 6 National Cancer Institute, National Institutes of Health, Rockville, MD 20850, USA; doddk@mail.nih.gov

**Keywords:** dietary supplements, infant, child, adolescent, income, food security, SNAP, WIC

## Abstract

This analysis characterizes use of dietary supplements (DS) and motivations for DS use among U.S. children (≤18 years) by family income level, food security status, and federal nutrition assistance program participation using the 2011–2014 National Health and Nutrition Examination Survey data. About one-third (32%) of children used DS, mostly multivitamin-minerals (MVM; 24%). DS and MVM use were associated with higher family income and higher household food security level. DS use was lowest among children in households participating in the Supplemental Nutrition Assistance Program (SNAP; 20%) and those participating in the Special Supplemental Nutrition Assistance Program for Women, Infants, and Children (WIC; 26%) compared to both income-eligible and income-ineligible nonparticipants. Most children who used DS took only one (83%) or two (12%) products; although children in low-income families took fewer products than those in higher income families. The most common motivations for DS and MVM use were to “improve (42% or 46%)” or “maintain (34 or 38%)” health, followed by “to supplement the diet (23 or 24%)” for DS or MVM, respectively. High-income children were more likely to use DS and MVM “to supplement the diet” than middle- or low-income children. Only 18% of child DS users took DS based on a health practitioner’s recommendation. In conclusion, DS use was lower among children who were in low-income or food-insecure families, or families participating in nutrition assistance programs.

## 1. Introduction

Dietary supplement (DS) use is widespread in the United States. More than half of adults [1,2] and approximately one-third of infants, children, and adolescents (henceforth children) use DS [3]. The use of DS is associated with socioeconomic status indicators such as family income level and food security in adults [4,5,6] and children [7,8,9,10]. For example, data from the 2007–2010 National Health and Nutrition Examination Survey (NHANES) demonstrated that children using DS tended to have higher income [9]. In a study using the 1999–2004 NHANES data, children using micronutrient supplement were more likely to have higher food security [8]. However, whether the type of DS used and motivations for their use differ by socioeconomic status remain unclear.

In the U.S., federal nutrition assistance programs such as the Supplemental Nutrition Assistance Program (SNAP) and the Special Supplemental Nutrition Assistance Program for Women, Infants, and Children (WIC) serve many low-income individuals or households with the goal of reducing food insecurity and nutritional risk [11]. These programs are targeted to improve the food and nutrition resources of participants. However, little is known about DS use of these nutrition assistance program participants [7,8,12], especially how they differ from income-eligible nonparticipants.

This analysis characterized DS use and examined motivations for use of DS among U.S. children aged 18 years and younger by family income level, food security status, and SNAP and WIC participation status using the most recent 2011–2014 NHANES data sets.

## 2. Materials and Methods

### 2.1. Study Design, Population, and Setting

The NHANES is a nationally representative, cross-sectional survey that samples U.S. noninstitutionalized civilians using a complex multistage probability sampling design [13]. The present analysis combined the 2011–2012 and 2013–2014 NHANES data of children (≤18 years), excluding those with missing DS use data. The final analytic sample was *n* = 8,288. All participants or their proxies provided written informed consent, and the Research Ethics Review Board at the National Center for Health Statistics (NCHS) approved the survey protocol.

The NHANES protocol includes an in-home interview and a physical examination in a mobile examination center. During the in-home interview, a proxy provided information for survey participants who were under 16 years of age. Demographic, socioeconomic, and lifestyle information was collected via computer-assisted software in the home interview. Age groups were aligned with the Dietary Reference Intakes (DRI) age groupings: <1, 1–3, 4–8, 9–13, and 14–18 years of age. Self-reported race and Hispanic origin groups as defined in the NHANES are non-Hispanic white, non-Hispanic black, non-Hispanic Asian, Hispanic, and other races; the “other” race group was only included in the estimates for the total sample as recommended [13]. Education level of the “household reference person,” defined as an adult household member who owns or rents the residence, was used to indicate the household’s education level. Education was categorized as less than high school, high school graduate or general equivalency diploma, some college or associate degree, and college graduate or above. Health insurance was categorized as none, private, or public; public health insurance included Medicaid, Children’s Health Insurance Program, state-sponsored or other government-sponsored health plan, and military health care [14]. Screen time was calculated as the sum of the total time spent looking at a television and/or computer screen per day for those aged ≥2 years (*n* = 4,006) using the Physical Activity Questionnaire. The response of “<1 h” was assigned 0.5 hours as recommended [15] and screen time was categorized as follows: ≤1, >1–≤2, >2–≤4, and >4 h/day. The American Academy of Pediatrics recommends limiting leisure screen time to two hours or less a day [16].

Family income was represented by the family income-to-poverty ratio (PIR), a ratio of family income to the poverty guideline established by the Department of Health and Human Services. The poverty guidelines are updated every year and vary by family size and geographic location (48 contiguous states, the District of Columbia, Alaska, and Hawaii) [17]. We categorized family income as PIR ≤ 130%, 131–350%, and >350% because a PIR of ≤130% is used as an eligibility criterion for several federal food assistance programs such as the Supplemental Nutrition Assistance Program (SNAP). Household food security status was measured using the U.S. Food Security Survey Module; an adult responded to 18 items for households with children. Households with more than three affirmative responses were categorized as food-insecure [18]. SNAP participation status was also collected at household level with the question, ‘‘Do you/does any member of your household currently receive SNAP or Food Stamp benefits?’’ and categorized as current participants, income-eligible nonparticipants (PIR ≤ 130%), and income-ineligible nonparticipants (PIR > 130%). WIC participation status was collected at individual level with the question “Is participant now receiving benefits from the WIC program?” and classified as current participants, income-eligible nonparticipants (PIR ≤ 185%), and income-ineligible nonparticipants (PIR > 185%).

During the household interview, detailed information about DS use during a 30-day period prior to the interview was collected using a product inventory, Dietary Supplement Questionnaire (DSQ). Participants or proxies were asked if they had taken any DS, and trained interviewers recorded each supplement’s name and manufacturer from the label, if available, or from the participant’s verbal report. Trained nutritionists at NCHS reviewed incoming data, obtained product labels, and incorporated DS information from the label into a database, including the name, ingredients, and product form. Data on the products participants reported and questions from the DSQ, along with product-level information from the labels, are all available on the NHANES website. For this analysis, DS were categorized into mutually exclusive categories based on their nutrient contents as published in a previous study [4]: (i) multivitamin and minerals (MVM) defined as a single product containing three or more vitamins and at least one mineral; (ii) multivitamins as a single product containing two or more vitamins without minerals; (iii) calcium-containing supplements (calcium as the primary ingredient with or without vitamin D or other nutrients); (iv) single-nutrient supplements, such as vitamin C, vitamin D, or iron; (v) botanicals; and (vi) fatty acids (any products with omega-3 or omega-6 fatty acids as the primary ingredient). The specific types of products shown by interviewers were selected based on high frequency of use; only the top products were reported. The reasons for taking each dietary supplement were also collected using a hand card with a list of reasons identified in previous surveys; participants were also able to provide other reasons not specified in the list and could choose more than one reason for each product. In addition, participants were asked if they used the supplement on their own or based on the advice of a doctor or other health practitioner.

During the physical examination, trained health technicians measured weight and height. Percentiles of body mass index (BMI) were used to categorize each participant’s weight status as underweight (<5th percentile), healthy weight (5th–85th percentiles), overweight (85th–95th percentiles), and obese (≥95th percentile) according to the growth charts developed by the Centers for Disease Control and Prevention (2000) for children 2–18 years; weight status was only available for children who attended the physical examination (*n* = 6,606). 

### 2.2. Statistical Analysis

Data were analyzed using SAS (version 9.4; SAS Institute, Inc, Cary, NC, USA) and SAS-callable SUDAAN (version 11; RTI International, Research Triangle Park, NC, USA) software programs. The 2011–2014 NHANES 4-year sample weights were used to account for differential probabilities of selection, nonresponse, and planned oversampling of some groups for all analyses. Interview weights were used for all analyses, except for the weight status analysis that used examination weights. Standard errors (SE) were estimated using a Taylor series linearization method. The statistical reliability of estimates were determined based on the relative standard error as recommended by NCHS [19]. Estimates with a relative SE > 30% may be statistically unreliable, so those with a relative SE > 30% and ≤40% were noted and those with the relative SE > 40% were not presented. Numbers of DS taken and motivations for DS use are estimated only from those who used DS in a 30-day period prior to the home interview (*n* = 2,365). We used pairwise t-tests to examine statistical significance of differences in categorical variables. To test for linear trends in ordinal variables, the null hypothesis of a nonlinear trend was examined with orthogonal polynomial contrasts. Statistical significance was determined at a Bonferroni-corrected *p*-value < 0.0167.

## 3. Results

In 2011–2014, an estimated 32% of children used DS in a 30-day period, with little difference by sex (Table 1). Infants (<1 year) were the least likely to use DS (16%). When infants were excluded, there was a significant trend toward lower DS use with increasing age. DS use was higher in non-Hispanic white (38%) and non-Hispanic Asian children (42%) compared to non-Hispanic black (21%) and Hispanic children (23%). Children with private health insurance (40%) were more likely to use DS than those with public (24%) or no health insurance (28%). The household’s education level was positively associated with DS use; whereas screen time in both boys and girls and weight status in girls were inversely associated with DS use.

When stratified by DRI age group, infants <1 year had distinct patterns of DS use compared to other age groups (Table S1). About a half of infant DS users were taking vitamin D, and 30% and 11% were using multivitamins and MVM, respectively. When infants were excluded, the proportion of MVM decreased with increasing age; older children tended to have greater diversity in terms of product type. Most used only one product, but the mean number of products used increased with age.

The most popular products used by children were the MVM (24%), followed by multivitamins (3.1%), vitamin C (2.4%), and vitamin D (1.6%) (Table 2). There were significant linear trends toward higher use of any DS, MVM, multivitamins, and vitamin D supplements with higher income (i.e., PIR). Use of any DS, MVM, and vitamin D supplement was also higher among children in food-secure than those in food-insecure households. Children in SNAP-participating households were least likely to use DS (20%) compared to either those in income-eligible (28%) or income-ineligible (40%) nonparticipating households in SNAP. Similarly, DS use was lowest in infants and young children participating in WIC (26%), compared with either income-eligible (36%) or income-ineligible nonparticipants in WIC (47%). Among children who used DS, the majority took one (83%) or two (12%) products (Table 3). The mean number of supplements taken was lower for low-income children (mean 1.15) than in either middle (mean 1.30) or higher-income (mean 1.30) children using the PIR criterion, but did not differ by SNAP or WIC participation and food security status among income-eligible children. 

**Figure 1:**
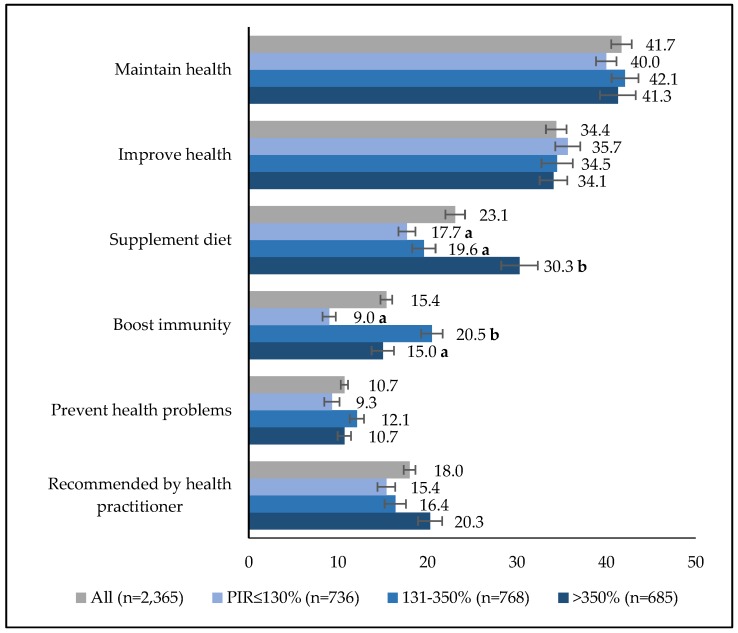
Estimated percentage (%(SE)) of DS users (≤18 years) who had the listed motivations for any dietary supplement use in a 30-day period by family income level, NHANES 2011–2014. PIR, family income-to-poverty ratio. Estimates with different letter subscripts (i.e., a or b) are significantly different across PIR subgroups at *p* < 0.0167. Participants could select more than one motivation for each product.

The top five motivations for DS use were “to improve overall health (42%),” “to maintain health (34%),” “to supplement the diet (23%),” “to prevent colds, boost immunity (15%),” and “to prevent health problems (11%)” (Figure 1). Most DS use was self-directed; only 18% of children were taking at least one product under the recommendation of a health care practitioner. Children in high-income families (i.e., PIR > 350%) were more likely to use DS “to supplement the diet” than those in middle- or low-income families. Among income-eligible children, motivations for DS use were not different by SNAP or WIC participation status. There were no significant differences in motivations for DS use by food security status (Table S2). Motivations for MVM use indicated that the high-income group was more likely to use MVM “to supplement diet” than were their lower-income counterparts (Table 4). The percentage of children who were using MVM at the recommendation of a health practitioner among SNAP participants (21%) was significantly higher than that of income-eligible non-SNAP (9%) children and income-ineligible nonparticipants (15%). The percentage of those who were using MVM due to health care provider’s recommendations was similar between WIC participants (26%) and income-eligible nonparticipants (22%).

## 4. Discussion

The prevalence of DS use among U.S. children has remained relatively consistent over time; about a third of U.S. children use DS, mostly micronutrient supplements such as MVM and multivitamins. Children in households with low incomes and food insecurity were less likely to use DS than those in more affluent households, as suggested in previous studies [7,8,9,10]. In addition, DS users in low-income families took fewer products and were less likely to have “supplementing the diet” as the motivation for their use of DS and MVM than those in higher-income families, even though low-income families may face barriers to nutrient-dense diets [20,21,22,23,24]. The impact of income differences in DS use on total nutrient intakes (i.e., nutrient intake from foods, fortification, and DS) among children should be further investigated, but results from adult NHANES data analysis suggested that income differences in DS use lead to larger disparities in total nutrient intake than when only nutrient intakes from foods are calculated [6,25].

DS use was lowest among children currently receiving WIC benefits (26%) and those in households receiving SNAP benefits (20%). This may be because the programs are linked to income. It is also notable that SNAP and WIC did not permit the purchase of DS with program benefits. Another possible explanation can be that the programs increase the amount of resources available for buying food, which may have eased parents’ concerns about the adequacy of their children’s diets. Previous studies also suggest that children receiving nutrition assistance (e.g. WIC, SNAP, and reduced/free school meals) were less likely to use any DS or nutrient supplements than nonparticipants of these nutrition assistance programs [7,8]. However, these studies did not further divide nonparticipants into income-eligible and income-ineligible nonparticipants, making it difficult to distinguish the effect of family income from that of nutrition assistance program participation. Nevertheless, USDA reports based on NHANES data have shown that DS use is lowest among SNAP and WIC participating children compared to both income-eligible and higher-income nonparticipants [26,27].

The proportion of the products taken by child users of DS on the basis of health care professional’s recommendations were about 16% in 2007–2010 survey [9]. Overall, 18% of children who used DS and 15% of those who used MVM took at least one product based on health practitioners’ recommendations. SNAP and WIC participants were more likely to use DS based on the recommendations of a health practitioner than income-eligible nonparticipating counterparts. WIC provides health care referrals in addition to food vouchers, and those referrals may have increased the program participant’s access to health practitioner’s recommendations [28,29] that may have served as cues for action [8].

Although DS are defined to supplement the diet under the Dietary Supplement Health and Education Act, four of the top five motivations for DS or MVM use were related to health promotion and disease prevention: “to maintain health,” “to improve overall health,” “to prevent colds,” and “to prevent health problems”. Our results supported that many DS users perceive supplements as “insurance” against health problems [30,31,32], although evidence of the health benefits of dietary supplements are controversial and complex [33,34,35]. DS use information of children aged <16 y was given by their proxies, so some motivations reported may be of parents or caregivers, not of children themselves. Moreover, it is possible that even motivations of children themselves were determined or largely influenced by parents and caregivers. Further research is needed on how parent’s perceptions and use of DS affect children’s DS use. So far, Yu et al. [36] reported that DS use of preschoolers was associated with mother’s supplement use before pregnancy and the mother’s perception of child’s eating behavior, and Dwyer et al. [37] showed that child DS use is similar to parents’ use in terms of type of product type. 

The characteristics of supplement users in our study were consistent with previous reports. Users tended to be younger (1–3 years and 4–8 years), be non-Hispanic white, have private health insurance, spend less time in front of television or computer screens, and have lower BMI (girls only) [7,8,9,10]. Because NHANES 2011–2014 oversampled non-Hispanic Asian persons, this study is the first to report estimates for non-Hispanic Asian children using NHANES data. The prevalence of DS use in non-Hispanic Asians was similar to that in non-Hispanic whites. The trends in use by sex, age, and weight status among children were different than those found in adults. In adults, DS use was higher in women, increased linearly with age, and was greatest among normal weight and lowest among underweight and obese adults [1,4]. Child DS users also had different patterns regarding the number and type of DS taken compared with adult users. The vast majority of child DS users (83%) were taking only one product, while 5% were taking 3 or more products; whereas about half of adult users took only one product and about 10% were using 5 or more products [38].

MVM were the most commonly used DS products across all age groups except infants (<1 year). Among infants, vitamin D as single-ingredient product was most frequently used. After infancy, the products used were more diverse in older children, similar to a greater variety of products used by adults [1,4]. For some age-sex groups, there may be a need for products containing certain ingredients. For example, under-consumption of iron by female adolescents was noted in the 2015–2020 Dietary Guidelines for Americans [39]. However, only 9.0% (SE 0.7%) took any iron-containing supplements (data not shown); this estimate was much lower than the estimate of 13.8% (SE 0.7%) for 14–18-year-old girls from a 1999–2002 NHANES analysis [7].

A limitation of our analysis was that DS use information of children under 16 years was obtained mainly from proxies who may not remember or observe whether their children actually consumed the product or how much they consumed. Another limitation is that the motivations for DS use were assessed at one point in time due to cross-sectional nature of the NHANES. Strengths of our study include the large nationally representative sample of children and rigorous methods used for DS information collection: in-person interview, checking containers and labels in home, and post hoc review and classification of the information by nutritionists. NHANES has collected information about motivations for DS use since 2007. To our knowledge, this study is the first to examine the detailed DS use information and motivations for DS use by economic indicators. 

There are many theories regarding DS use. Some of our results highlight the “inverse supplement hypothesis” that suggests healthier and more health-conscious people with better quality diets are more likely to use DS [30,31], and the Health Belief Model that suggests limited financial resources may be one of the modifying factors that may supersede intentions for DS use [8]. Nutrients from DS can contribute substantial amount of nutrients to total nutrient intake and, therefore, may fill the nutrient gaps for those who otherwise would not meet recommended intake targets of some nutrients [40,41,42]. DS may differentially contribute to total nutrient intake by socioeconomic status as shown in previous studies using PIR as a poverty indicator [6,25]. However, this argument assumes that the nutrient gaps are filled by the DS taken, which is highly dependent on whether there is a nutrient gap to begin with and whether DS taken contain the deficient nutrient. Future work should estimate total nutrient intakes from foods and DS to identify the proportions of various socioeconomic subpopulations that are not meeting Estimated Average Requirements or exceeding Tolerable Upper Intake Levels and to what extent nutrients from DS contribute to total intakes. At the same time, more investigations on the safety of DS and the efficacy of DS are needed. The practical implementation and behavioral changes necessary for effective supplement use among children in households with limited resources are also unknown. More efforts are warranted to ensure adequate nutrition across all socioeconomic groups, taking into account the complex interplay of socioeconomic, lifestyle, health, and psychological determinants and incorporating diverse actors, including caregivers, health care providers, and society.

## 5. Conclusions

DS are used by about a third of U.S. children, with most child DS users using MVM or multivitamins and taking only one product in a 30-d period. DS use was greater among children in families with a higher household income and a higher level of household food security, and was lowest among children living in lower income families who were participating in WIC or SNAP. The most common motivations for DS use were related to health across all subgroups, while children in high-income families were more likely to use DS “to supplement the diet.” The data suggest that there are systematic differences in DS use and types of DS used by family income level, food security level, and federal nutrition assistance program participation status.

## Figures and Tables

**Table 1:** Estimated percentage of U.S. children (≤18 years) who used any dietary supplement in a 30-day period by demographic, lifestyle, and anthropometric characteristics, NHANES 2011–2014 ^1^.

Characteristic	All		Male		Female	
	*n*	% (SE)	*n*	% (SE)	*n*	% (SE)
Total	8,288	32.4 (1.2)	4,217	32.2 (1.35)	4,071	32.7 (1.46)
Age						
<1 year	797	16.4 (1.5)	394	16.4 (2.4)	403	16.5 (2.2)
1–3 years	1,537	38.6 (2.4)	768	39.0 (3.0)	769	38.3 (3.1)
4–8 years	2,265	39.4 (1.7)	1,196	38.5 (2.2)	1,069	40.5 (2.3)
9–13 years	1,995	30.9 (1.5)	1,008	30.5 (1.9)	988	31.2 (2.4)
14–18 years	1,694	26.3 (1.7) *	851	26.0 (2.7) *	843	26.5 (2.4) *
Race/Ethnicity ^2^						
Non-Hispanic white	2,055	38.1 (1.7) ^a^	1,073	38.2 (2.2) ^a^	982	37.9 (2.3) ^a^
Non-Hispanic black	2,221	21.2 (1.6) ^b^	1,147	21.2 (1.9) ^b^	1,074	21.2 (1.5) ^b^
Non-Hispanic Asian	869	41.7 (3.2) ^a^	434	38.9 (3.9) ^a^	435	44.8 (3.1) ^a^
Hispanic	2,632	22.8 (1.1) ^b^	1,311	21.7 (1.4) ^b^	1,321	24.1 (1.6) ^b^
Health Insurance						
Private	3,255	40.0 (1.7) ^a^	1,639	41.3 (2.2) ^a^	1,616	38.8 (2.2) ^a^
Public	4,321	24.1 (1.3) ^b^	2,219	22.7 (1.5) ^b^	2,102	25.7 (1.6) ^b^
None	663	27.5 (2.7) ^b^	337	26.7 (4.3) ^b^	326	28.5 (2.7) ^b^
Household’s Education Level						
Less than high school	2,014	18.6 (1.6)	1,044	19.6 (2.3)	970	17.6 (1.8)
High school grad/GED or equivalent	1,822	25.1 (1.4)	928	24.9 (2.1)	894	25.2 (1.8)
Some college or associate degree	2,344	32.7 (1.9)	1,192	32.6 (2.3)	1,152	32.9 (2.1)
College graduate or above	1,823	47.1 (2.2) *	910	45.7 (2.5) *	913	48.5 (3.2) *
Screen Time (≥2 years)						
≤ 1 h/day	1,120	37.7 (2.8)	511	36.0 (2.9)	609	39.1 (3.5)
>1–≤ 2 h/day	1,477	36.9 (1.9)	709	38.7 (2.9)	768	35.1 (2.4)
>2–≤ 4 h/day	2,527	34.9 (1.6)	1,328	33.8 (2.2)	1,199	36.0 (2.3)
>4 h/day	1,753	26.5 (1.2) *	959	26.4 (2.1) *	794	26.6 (2.1) *
Weight Status (≥2 years) ^3^						
Underweight	239	38.2 (4.3)	130	33.6 (5.4)	109	43.4 (6.3)
Normal Weight	4,171	36.9 (1.5)	2,119	36.8 (1.6)	2,052	37.0 (1.9)
Overweight	1,028	31.2 (2.0)	530	32.0 (3.5)	498	30.5 (2.9)
Obese	1,168	24.5 (1.8) *	599	25.0 (2.4)	569	24.0 (2.5) *

Abbreviations: GED, general equivalency diploma. ^1^ Estimates with different letter subscripts (i.e., a or b) are significantly different across subgroups within each category at *p* < 0.0167; asterisk (*) indicates significant linear trend at *p* < 0.0167. For age comparison, infants <1 year were not included in the contrast. ^2^ “Other” race group (*n* = 259) was not presented as recommended by the National Center for Health Statistics (NCHS).^3^ Data were examined separately using the examination weight.

**Table 2:** Estimated percentage of U.S. children (≤18 years) who used dietary supplement in a 30-day period by economic indicators, NHANES 2011–2014 ^1,2^.

Characteristic	*n*	Any DS	MVM	Multivitamins	Vitamin C	Vitamin D
		% (SE)				
Total	8,288	32.4 (1.2)	24.1 (1.2)	3.1 (0.5)	2.4 (0.3)	1.6 (0.2)
PIR						
≤130%	3,726	22.2 (1.6) ^a^	17.1 (1.4) ^a^	1.8 (0.4) ^a^	1.1 (0.3) ^a^	0.9 (0.2) ^a^
131–350%	2,379	34.6 (1.5) ^b^	24.4 (1.6) ^b^	4.0 (0.9) ^a,b^	3.2 (0.7) ^b^	1.7 (0.4) ^a,b^
>350%	1,533	44.7 (2.4) ^c,^*	33.6 (3.0) ^c,^*	4.0 (0.8) ^b,^*	3.3 (0.9) ^a,b^	2.7 (0.6) ^b,^*
Food security						
Food-insecure	2,169	22.3 (2.0) ^a^	15.8 (1.3) ^a^	2.8 (0.8)	1.9 (0.6)	0.4 (0.1) ^a,2^
Food-secure	6,055	35.1 (1.3) ^b^	26.2 (1.5) ^b^	3.2 (0.5)	2.5 (0.4)	2.0 (0.3) ^b^
SNAP participation						
Participant	2,922	19.5 (1.5) ^a^	14.4 (1.3) ^a^	1.6 (0.5) ^a^	0.6 (0.2) ^a,2^	0.8 (0.3) ^a,2^
Income-eligible nonparticipant	1,377	27.9 (1.9) ^b^	22.4 (1.9) ^b^	1.6 (0.5) ^a^	2.0 (0.7) ^a,b,2^	0.8 (0.3) ^a,2^
Income-ineligible nonparticipant	3,509	40.3 (1.6) ^c^	29.3 (1.9) ^c^	4.3 (0.8) ^b^	3.5 (0.5) ^b^	2.3 (0.4) ^b^
WIC participation						
Participant	1,562	25.9 (1.8) ^a^	19.4 (1.7) ^a^	3.3 (0.8)	—	0.8 (0.3) ^a,2^
Income-eligible nonparticipant	386	35.5 (3.9) ^b^	28.6 (3.7) ^a,b^	4.1 (1.4) ^2^	—	—
Income-ineligible nonparticipant	764	47.1 (2.3) ^c^	33.7 (3.0) ^b^	5.2 (1.0)	1.1 (0.4) ^2^	3.7 (0.7) ^b^

Abbreviations: DS, dietary supplement; MVM, multivitamin-minerals; NHANES, National Health and Nutrition Examination Survey; PIR, family income-to-poverty ratio; SNAP, Supplemental Nutrition Assistance Program; WIC, the Special Supplemental Nutrition Assistance Program for Women, Infants, and Children. ^1^ Estimates with different letter subscripts (i.e., a, b, or c) are significantly different across subgroups within each indicator category at *p* < 0.0167; asterisk (*) indicates significant linear trend across PIR subgroups at *p* < 0.0167; ^2^ The relative SE is >30% but ≤40% and may be statistically unreliable. If the relative SE > 40%, data are not shown (—).

**Table 3:** Estimated percentage distribution and mean number of any dietary supplement taken by U.S. children (≤18 years) in a 30-day period by economic indicators, NHANES 2011–2014 ^1,2^.

	All (*n* = 2,365)	PIR (*n* = 2,189)			Food security (*n* = 2,339)		SNAP Participation (*n* = 2,220)			WIC Participation (<5 years; *n* = 793)		
		≤130% (*n* = 736)	130–350% (*n* = 768)	>350% (*n* = 685)	Food-Insecure (*n* = 443)	Food-Secure (*n* = 1,896)	Participant (*n* = 532)	Income-Eligible Non-SNAP (*n* = 344)	Income-Ineligible Non-SNAP (*n* = 1,344)	Participant (*n* = 339)	Income-Eligible Non-WIC (*n* = 113)	Income-Ineligible Non-WIC (*n* = 341)
Number of supplements taken, % (SE)												
1	82.7 (1.5)	89.1 (2.1) ^a^	81.9 (2.6) ^b^	78.9 (2.8) ^b,^*	81.8 (1.7)	87.2 (2.5)	91.3 (1.9) ^a^	86.7 (3.6) ^a,b^	79.9 (1.9) ^b^	92.2 (3.0)	95.2 (2.5)	86.6 (3.0)
2	11.9 (1.1)	7.6 (1.5) ^a^	12.0 (2.1) ^a,b^	14.8 (2.0) ^b,^*	12.5 (1.2)	9.0 (2.3)	5.9 (1.7) ^a^	10.0 (2.5) ^a,b^	13.6 (1.6) ^b^	—	—	12.0 (3.0)
3 or more	5.4 (0.8)	3.3 (1.3) ^2^	6.1 (1.4)	6.3 (1.5)	5.7 (0.9)	—	—	—	6.4 (0.9)	—	—	—
Mean number of supplements taken, mean (SE)	1.26 (0.03)	1.15 (0.03) ^a^	1.30 (1.3) ^b^	1.30 (0.04) ^b,^*	1.28 (0.03)	1.18 (0.04)	1.13 (0.03) ^a^	1.17 (0.05) ^a^	1.31 (0.03) ^b^	1.09 (0.03)	1.06 (0.03)	1.16 (0.03)

Abbreviations: NHANES, National Health and Nutrition Examination Survey; PIR, family income-to-poverty ratio; SNAP, Supplemental Nutrition Assistance Program; WIC, the Special Supplemental Nutrition Assistance Program for Women, Infants, and Children. ^1^ Estimates with different letter subscripts (i.e., a or b) are significantly different across subgroups within each indicator category at *p* < 0.0167; asterisk (*) indicates significant linear trend across PIR subgroups at *p* < 0.0167. ^2^ The relative SE is >30% but ≤40% and may be statistically unreliable. If the relative SE > 40%, data are not shown (—).

**Table 4:** Estimated percentage (%(SE)) of multivitamin-mineral users (≤18 years) and motivations for use in a 30-day period by economic indicators, NHANES 2011–2014 ^1,2^.

	All (*n* = 1,716)	PIR (*n* = 1,588)			Food Security (*n* = 1,694)		SNAP Participation (*n* = 1,609)			WIC Participation (<5 years; *n* = 544)		
		≤130% (*n* = 537)	130–350% (*n* = 543)	>350% (*n* = 508)	Food-Insecure (*n* = 314)	Food-Secure (*n* = 1,380)	Participant (*n* = 387)	Income-Eligible Non-SNAP (*n* = 254)	Income-Ineligible Non-SNAP (*n* = 968)	Participant (*n* = 225)	Income-Eligible Non-WIC (*n* = 84)	Income-Ineligible Non-WIC (*n* = 235)
**Top 5 motivations**												
**To maintain health**	45.7 (2.3)	44.0 (3.2)	45.0 (4.2)	46.0 (4.2)	43.1 (4.1)	45.8 (2.8)	45.5 (3.9)	43.9 (4.9)	45.4 (3.4)	43.0 (4.2)	50.0 (5.7)	41.7 (4.8)
**To improve overall health**	38.0 (2.9)	41.5 (3.5)	38.7 (4.1)	35.5 (3.8)	42.1 (5.5)	37.4 (3.1)	40.6 (4.3)	39.7 (5.5)	37.2 (3.6)	31.0 (4.6)	43.6 (10.0)	40.6 (4.0)
**To supplement diet**	23.9 (2.6)	17.1 (2.4) ^a^	19.9 (2.9) ^a^	32.3 (4.6) ^b,^*	18.6 (3.1)	24.7 (3.1)	15.3 (2.7) ^a^	18.4 (3.1) ^a,b^	27.2 (3.6) ^b^	26.9 (3.2)	22.5 (5.8)	29.9 (4.6)
**To prevent health problems**	10.4 (0.9)	10.0 (2.0)	12.8 (2.4)	8.6 (1.6)	9.5 (2.2)	10.7 (1.1)	10.0 (2.0)	10.4 (3.0)	10.8 (1.3)	13.1 (3.4)	13.0 (4.5) ^2^	9.0 (2.8) ^2^
**To prevent colds, boost immunity**	10.4 (1.0)	7.1 (1.3) ^a^	12.5 (2.0) ^b^	10.1 (1.8) ^b^	11.4 (2.5)	10.0 (1.1)	9.0 (1.9)	6.6 (1.8)	11.3 (1.6)	7.9 (2.1)	—	13.6 (3.3)
**Health practitioner recommended**												
**Yes**	15.3 (1.6)	12.4 (2.5)	13.9 (2.4)	17.2 (3.4)	15.1 (2.4)	15.0 (2.0)	20.5 (3.6) ^a^	8.7 (2.2) ^b^	15.0 (2.2) ^b^	26.4 (3.9)	—	21.8 (4.7)

Abbreviations: NHANES, National Health and Nutrition Examination Survey; PIR, family income-to-poverty ratio; SNAP, Supplemental Nutrition Assistance Program; WIC, the Special Supplemental Nutrition Assistance Program for Women, Infants, and Children. ^1^ Estimates with different letter subscripts (i.e., a or b) are significantly different across subgroups within each indicator category at *p* < 0.0167; asterisk (*) indicates significant linear trend across PIR subgroups at *p* < 0.0167. ^2^ The relative SE is >30% but ≤40% and may be statistically unreliable. If the relative SE > 40%, data are not shown (—).

## Supplementary Materials

The following are available online at http://www.mdpi.com/2072-6643/10/9/1212/s1, Table S1: Estimated percentage distribution and mean number of dietary supplement taken by U.S. children (≤18 years) in a 30-day period, by age group, NHANES 2011–2014, Table S2: Estimated percentage (%(SE)) of any dietary supplement users (≤18 years) and motivations for use in a 30-day period by food security and SNAP and WIC participation status, NHANES 2011–2014.
