# Supplementary material for: Dietary Supplement Use among U.S. Children by Family Income, Food Security Level, and Nutrition Assistance Program Participation Status in 2011–2014
Source: Nutrients. 2018 Sep 1;10(9):1212. doi: 10.3390/nu10091212 (PMC6163871; doi:10.3390/nu10091212)
Supplement: Supplementary file 1 [file nutrients-10-01212-s001.pdf]

Table S1. Estimated percentage distribution and mean number of dietary supplement taken by U.S. children (≤18 years) in a 30-d period, by age group, NHANES 2011–2014 [1,2].

Characteristic	All (*n* = 2,365)	<1 year (*n* = 119)	1-3 years (*n* = 509)	4-8 years (*n* = 800)	9-13 years (*n* = 519)	14-18 years (*n* = 419)
	% (SE)					
**Type or products**						
MVM*	74.3 (1.7)	10.6 (2.6)	78.5 (2.6)	82.6 (2.2)	76.1 (2.7)	62.7 (3.6)
Multivitamins	9.6 (1.4)	29.7 (4.7)	10.8 (1.8)	10.2 (2.2)	9.3 (1.9)	5.7 (1.4)
Vitamin C*	7.3 (1.0)	0.0 (0.0)	—	4.4 (1.1)	8.3 (1.6)	16.2 (3.0)
Vitamin D*	5.0 (0.7)	47.3 (6.2)	1.8 (0.7) [2]	2.7 (0.9) [2]	3.0 (0.7)	8.9 (2.3)
Calcium*	4.7 (0.7)	0.0 (0.0)	—	5.0 (1.3) [2]	3.6 (1.3) [2]	8.3 (2.2)
Botanicals*	4.2 (0.9)	—	—	—	—	8.3 (1.8)
Fatty acids*	2.4 (0.6)	0.0 (0.0)	—	—	1.4 (0.5) [2]	7.6 (2.6) [2]
Iron	1.6 (0.4)	—	1.2 (0.4) [2]	—	—	4.2 (1.2)
**Number of supplements taken, % (SE)**						
1*	82.7 (1.5)	91.8 (2.7)	90.1 (2.1)	85.6 (2.3)	82.9 (2.3)	71.0 (2.8)
2*	11.9 (1.1)	5.8 (2.1)	9.3 (2.0)	8.8 (1.6)	13.1 (1.9)	18.0 (2.3)
3 or more*	5.4 (0.8)	—	—	5.6 (1.4)	4.0 (1.1)	11.0 (2.4)
**Mean number of supplements taken, mean (SE)***	1.3 (0.03)	1.1 (0.04)	1.1 (0.02)	1.2 (0.04)	1.2 (0.04)	1.5 (0.08)

Abbreviations: MVM, multivitamin-minerals; NHANES, National Health and Nutrition Examination Survey.

[1] Asterisk (*) indicates significant linear trend across age groups at P < 0.0167. Infants <1 year were not included in the contrast. [2] The relative SE is >30% but ≤40% and may be statistically unreliable. If the relative SE>40%, data are not shown (—).

Table S2. Estimated percentage (%(SE)) of any dietary supplement users (≤18 years) and motivations for use in a 30-d period by food security and SNAP and WIC participation status, NHANES 2011–2014 [1,2].

	**Food security (*n* = 2,339)**		**SNAP participation (*n* = 2,220)**			**WIC participation (<5 years; *n* = 793)**		
	**Food-insecure (*n* = 443)**	**Food-secure (*n* = 1,896)**	**Participant (*n* = 532)**	**Income-eligible non-SNAP (*n* = 344)**	**Income-ineligible non-SNAP (*n* = 1,344)**	**Participant (*n* = 339)**	**Income-eligible non-WIC (*n* = 113)**	**Income-ineligible non-WIC (*n* = 341)**
**Top 5 motivations**								
To maintain health	41.3 (3.8)	41.6 (2.7)	41.0 (3.8)	39.3 (4.0)	41.7 (3.2)	39.1 (3.8)	46.8 (4.7)	38.1 (4.1)
To improve overall health	36.5 (4.9)	34.1 (2.6)	35.4 (3.6)	35.6 (4.4)	34.2 (3.0)	29.2 (3.8)	36.5 (8.3)	34.8 (3.2)
To supplement diet	16.9 (2.7)	24.2 (2.7)	16.3 (2.5)	18.5 (2.2)	25.8 (3.2)	24.0 (2.8)	22.1 (4.7)	29.9 (3.9)
To prevent health problems	14.8 (2.8)	15.4 (1.6)	11.1 (1.8) [a,b]	9.8 (2.4) [a]	17.6 (2.0) [b]	10.4 (2.5)	—	14.8 (2.7)
To prevent colds, boost immunity	9.6 (2.0)	11.1 (0.9)	9.6 (1.7)	9.9 (2.5)	11.4 (1.0)	12.1 (2.5)	12.1 (4.6) 2	8.2 (2.0)
**Health practitioner recommended**								
Yes	15.4 (2.0)	18.3 (1.6)	22.9 (3.0) [a]	10.8 (1.9) [b]	18.0 (1.8) [a,b]	30.9 (3.2) [a]	14.6 (3.5) [b]	28.9 (4.6) [a]

Abbreviations: NHANES, National Health and Nutrition Examination Survey; SNAP, Supplemental Nutrition Assistance Program; WIC, the Special Supplemental Nutrition Assistance Program for Women, Infants, and Children.

[1] Estimates with different letter subscripts (i.e., a or b) are significantly different across subgroups within each indicator category at P < 0.0167. [2] The relative SE is >30% but ≤40% and may be statistically unreliable. If the relative SE>40%, data are not shown (—).
